# Identifying the climatic drivers of honey bee disease in England and Wales

**DOI:** 10.1038/s41598-021-01495-w

**Published:** 2021-11-09

**Authors:** Ben W. Rowland, Stephen P. Rushton, Mark D. F. Shirley, Mike A. Brown, Giles E. Budge

**Affiliations:** 1 School of Natural and Environmental Sciences, Newcastle University, Ridley Building 2, Newcastle upon Tyne, NE1 7RU Tyne and Wear UK; 2 National Bee Unit, Animal and Plant Health Agency, Sand Hutton, York, YO41 1LZ UK

**Keywords:** Zoology, Environmental sciences

## Abstract

Honey bee colony health has received considerable attention in recent years, with many studies highlighting multifactorial issues contributing to colony losses. Disease and weather are consistently highlighted as primary drivers of colony loss, yet little is understood about how they interact. Here, we combined disease records from government honey bee health inspections with meteorological data from the CEDA to identify how weather impacts EFB, AFB, CBP, varroosis, chalkbrood and sacbrood. Using R-INLA, we determined how different meteorological variables influenced disease prevalence and disease risk. Temperature caused an increase in the risk of both varroosis and sacbrood, but overall, the weather had a varying effect on the six honey bee diseases. The risk of disease was also spatially varied and was impacted by the meteorological variables. These results are an important step in identifying the impacts of climate change on honey bees and honey bee diseases.

## Introduction

The European or Western honey bee (*Apis mellifera*) is a vital agricultural species in the UK, providing £150 million in pollination services to agriculture and producing £35 million of honey^1^. The UK managed honey bee populations have faced a decline in recent years^2^ due to multiple interacting pressures, including socio-economic changes, land-use intensification, agrochemical exposure and the impact of parasites/pathogens^3^.

Honey bees suffer from a range of bacterial, fungal, microsporidial and viral pathogens, as well as ectoparasitic mites, which can all lead to poor colony health and colony loss. Specifically, European foulbrood (EFB)^4^, American foulbrood (AFB)^5^, chronic bee paralysis (CBP)^6^, sacbrood^7^, chalkbrood^8^ and varroosis^9^ are all diseases that can have well documented direct adverse effects on honey bee colonies, such as brood deterioration and paralysis of adult bees. In addition to the obvious direct effects on colony health, disease can have more subtle indirect consequences that are less well defined and more difficult to study. For example, loss of workers due to colony infestation with *Varroa destructor* mites results in poor foraging and subsequent larval and adult starvation^10^. In addition, shortened lifespans of bees infected with the mite-associated deformed wing virus (DWV) can lead to winter colony losses^11^. Brood diseases such as AFB, EFB, sacbrood and chalkbrood are known to have significant direct effects on the health of colonies because of their impact on early life stages, later limiting the number of future workers available to conduct essential hive tasks^4,5^.

Honey bees meet their entire nutritional needs by foraging in their local environment for nectar (carbohydrate) and pollen (protein), but foraging is heavily dependent on climatic conditions. Rainfall, low temperatures and high winds are all known to restrict honey bee foraging activity. Pollen dearth can occur within a few days absence of forage^12^, leading to earlier larval capping^13^ and reduced nursing^14^. Consumption of non-fresh pollen can lead to gut dysbiosis and an increase in pathogen prevalence^15^. Poor weather in spring and summer, when colonies are most populous, can lead to increased hive congestion^14,16,17^, which has in turn been linked to an increase in disease transmission^18^. Predicted future climate change may bring more severe and unpredictable weather that could ultimately be detrimental to honey bee colony survival^19,20^ and may alter naturally available food sources^21^.

Identifying the key drivers of honey bee diseases is vital to preserving the health of honey bee populations which contribute directly and indirectly to global food security. It is important to know how natural drivers contribute to honey bee disease prevalence, given the changing global climate. In this study, we aim to (1) identify the spatial and temporal patterns of honey bee diseases between 2006 and 2016; (2) determine the most significant meteorological drivers of disease; and (3) determine how the risk of each disease varies with the weather, space and time.

## Results

**Figure 1:**
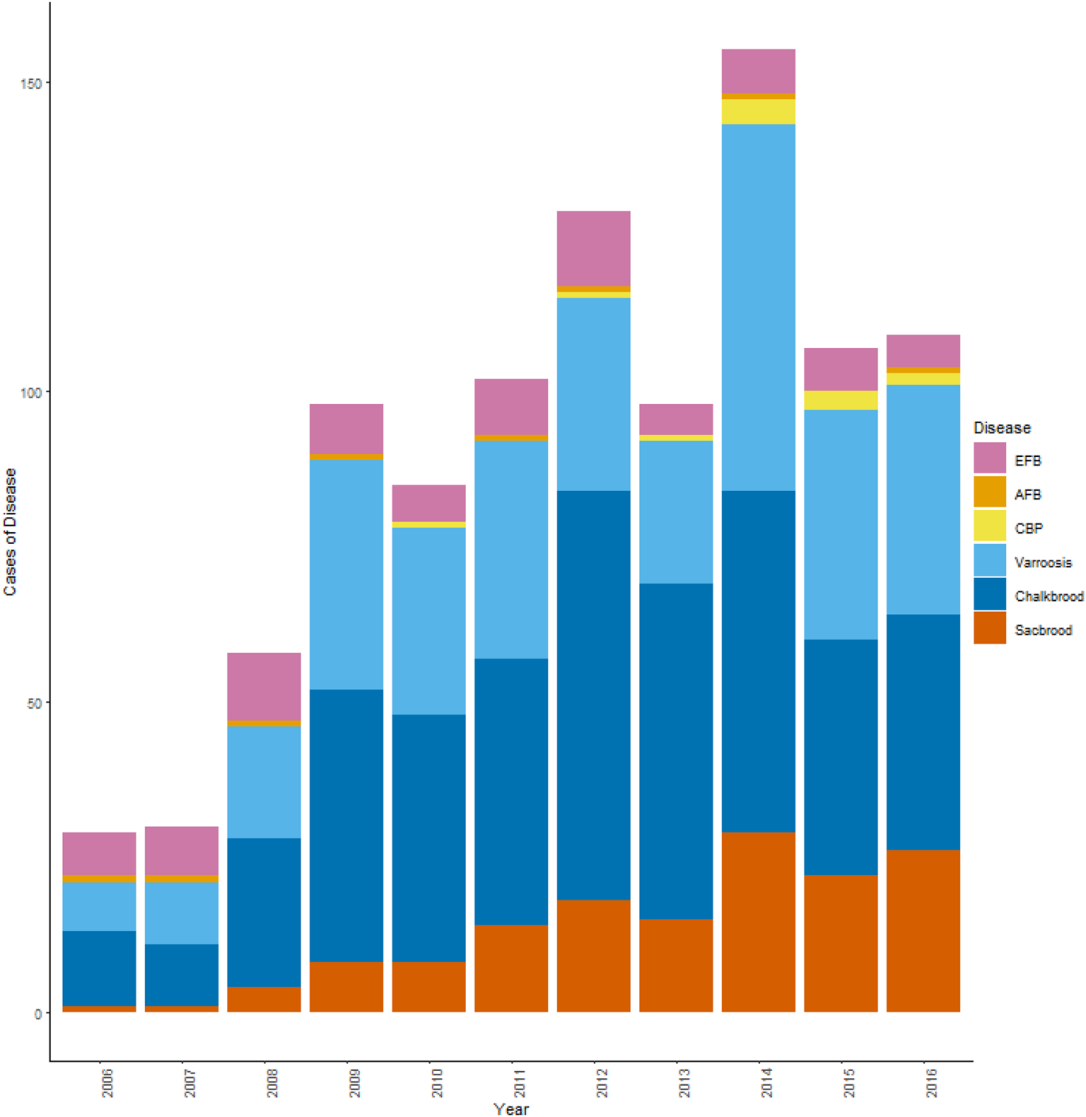
Annual number of cases for European Foulbrood (EFB), American Foulbrood (AFB), Chronic bee paralysis (CBP), varroosis, chalkbrood and sacbrood between 2006 and 2016. Cases of disease were normalised per 1000 colonies visited to avoid inspection bias.

Between 2006 and 2016, there were 317,838 visits to colonies by NBU inspectors, with an average of 28,894 per year. The prevalence of each disease varied yearly (Fig. 1). AFB was the rarest disease, followed by CBP and then EFB. Varroosis, sacbrood and chalkbrood were the three most prevalent diseases observed during the inspections (Fig. 1).

**Figure 2:**
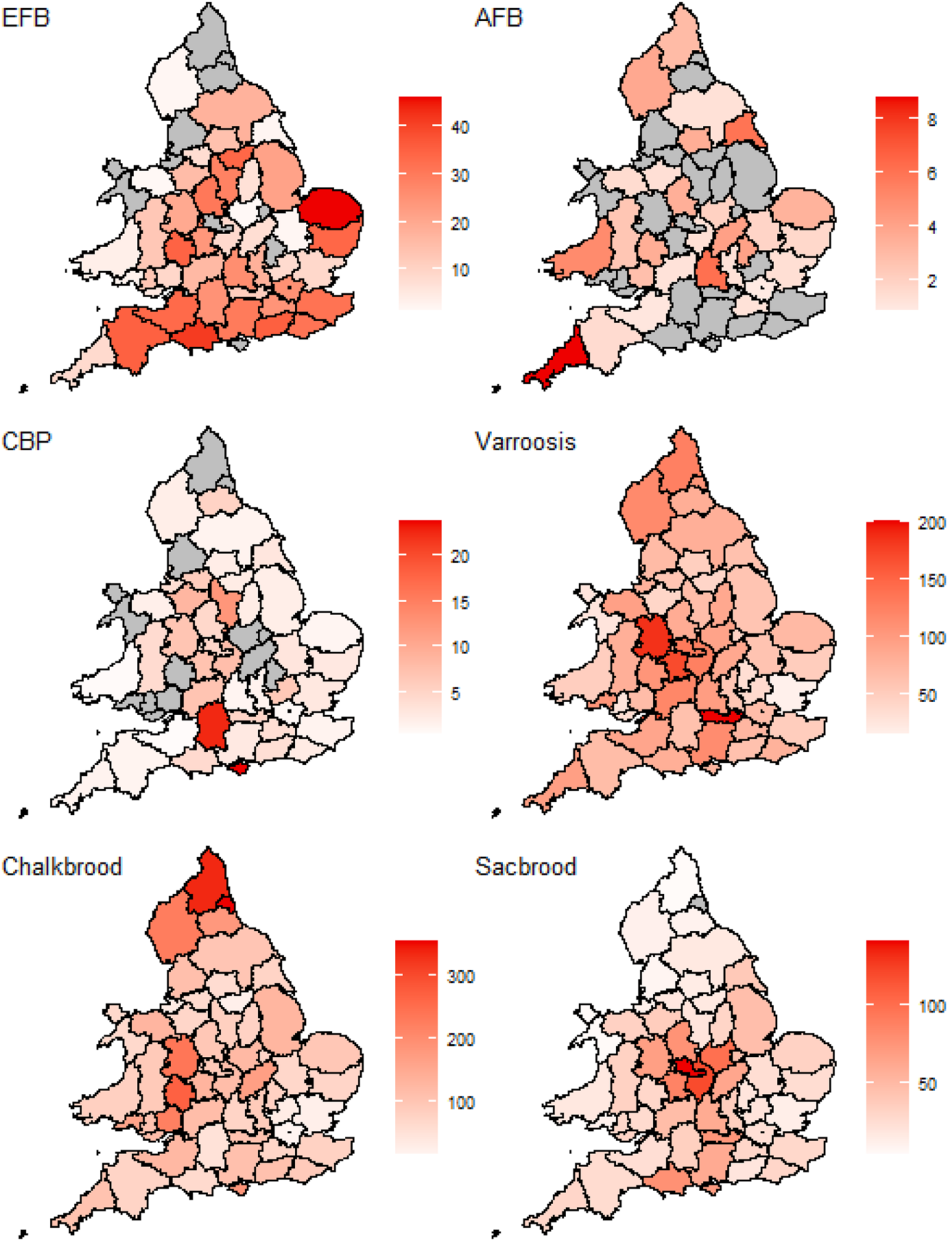
Total number of cases of different honey bee diseases in England and Wales between 2006 and 2016. Each county was normalized to cases per 1000 colonies visited to avoid inspection bias. To maintain confidentiality, data are greyed out when fewer than five apiaries were visited in a single county/year. The county boundaries for England and Wales were sourced from the Database of Global Administrative Areas (http://GADM.org).

All diseases appear to vary spatially, with each of the diseases varying in its prevalence across England and Wales and having different counties where they are more or less prevalent (Fig. 2). EFB, varroosis and chalkbrood appeared to be the most spatially abundant diseases.

### BYM models

**Figure 3:**
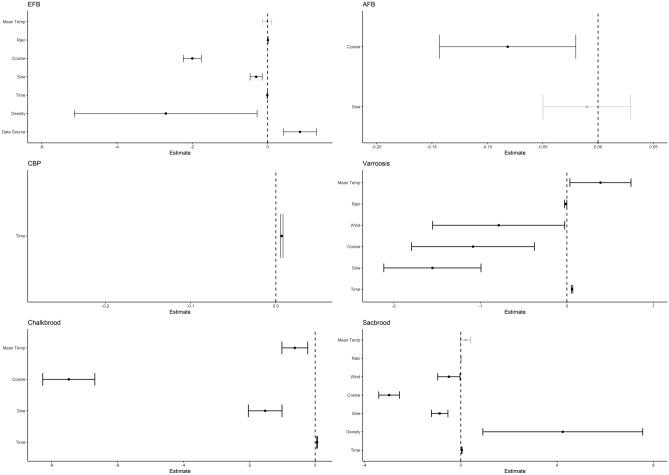
Fixed effects from the R-INLA models. The 97.5% and 2.5% confidence intervals and mean are shown for each significant fixed effect. Most nonsignificant effects were removed as part of the modelling process, and any remaining nonsignificant effects did not improve the R-INLA models and were left in and are displayed in grey.

The results of the BYM models indicated that climatic variables had significant effects on the spatio-temporal pattern of recorded bee disease in England, with the exception of CBP and AFB (Fig. 3). Incidence of EFB disease was significantly related to rain with a 0.9% (2.5% and 97.5% ci were 0.4% and 1.4%) increase in the risk of disease in a county per mm of rain. There was a reduction in the general risk of EFB over time with a monthly decrease in EFB risk of 0.4% (2.5% and 97.5% ci were − 0.7% and − 0.1%). The risk of AFB, in contrast, was not significantly affected by any weather variables and lacked any relationship with time. CBP has recently been described as an emerging disease^22^, so as expected, it had a significant relationship with time increasing in risk by 0.7% per month. Interestingly, CBP also had no significant relationships with any of the meteorological variables. For varroosis, mean temperature (38.9%) and time (6%) had a significant positive relationship per unit increase, and both rain (1.4%) and wind (77.2%) had a significant negative impact per unit increase. The relationship chalkbrood had with mean temperature (47.8%) was negative, with time being the only positive predictor indicating a 5.8% increase in disease risk per month. Lastly, sacbrood was significantly influenced by mean temperature (25.7%) and wind (57.7%), with the relationship being positive for temperature and negative for wind. Sacbrood was also significantly affected by time, having a positive relationship of a 5.2% increase in risk per month. The harmonic variables were all significant for EFB, AFB, varroosis, chalkbrood and sacbrood, indicating the importance of season in disease prevalence. The 2.5% and 97.5% credibility intervals for all the diseases are displayed in Supplementary Table S1 of the Supplementary Information. County-level apiary density had minimal effect on the risk of honey bee diseases with a large degree of error associated with the estimate, only negatively affecting the risk of EFB and positively affecting the risk of sacbrood virus. Similarly, the apiary density data source only had a significant effect on EFB.

Meteorological variables impacted diseases differentially, and the directionality of the relationship also varied (Fig. 3). The wind had the most significant effect on varroosis and sacbrood. Chalkbrood declined with increasing mean temperature, but varroosis and sacbrood increased. The harmonic variables sine and cosine had the greatest impact on varroosis and chalkbrood, showing the effect of season on disease prevalence and the importance of accounting for seasonal changes in the data. Regional apiary density was the largest impacting factor on EFB and sacbrood but had opposite effects.

**Figure 4:**
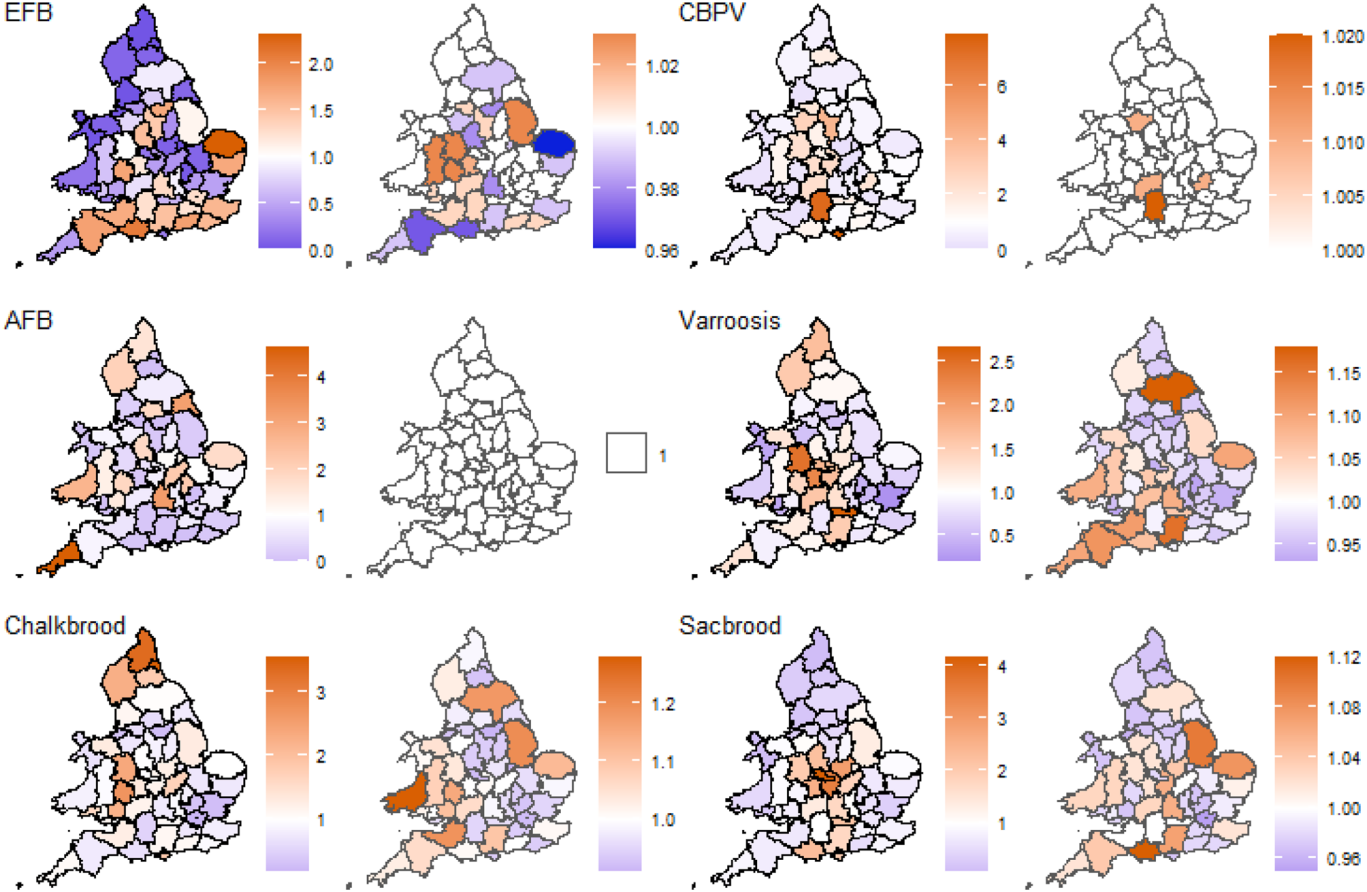
The predicted disease risk based on the raw inspection data (left) and the relative risk obtained from the R-INLA models (right). Orange indicates a higher than average risk, white indicates average risk and blue indicates lower than average risk. To maintain confidentiality, data are greyed out when fewer than five apiaries were visited in a single county/year. The county boundaries for England and Wales were sourced from the Database of Global Administrative Areas (http://GADM.org).

The spatial pattern of risk for most diseases varied across English and Welsh counties (Fig. 4). Figure 4 shows the true risk for each county based on the observed and expected number of cases and the risk of disease in individual counties compared to the rest of the country after accounting for the risk from impacting variables. It is important to note that the maps report relative risk across counties and are not necessarily showing direct changes in the amount of risk. That being said, the risk of EFB was very low in the northern counties and significantly higher along the southern coast. After accounting for weather, many counties risk values were equivalent to that rest of the country, while several counties in the south and east saw risk levels decrease after accounting for the weather. Counties in the Midwest saw an increase suggesting an increase in risk from weather. The risk of AFB remained relatively low, with only a few counties being significantly above or below normal risk levels. After accounting for the weather with all counties had the same level of risk as to the country as a whole. This is somewhat surprising given that weather did not have a significant impact on AFB in the R-INLA models. The risk maps for CBP appear almost identical, with the minimal risk being accounted for in the weather variables. Comparing the risk levels for varroosis and chalkbrood, they were remarkably similar both before and after accounting for the risk from the weather. Many counties saw the same trend in risk between the two maps. Lastly, the risk of sacbrood seemed to be confined primarily to the interior of the country. After accounting for weather, a few counties for some diseases saw an increase in the risk stemming from the negative relationship they had with the weather, but as the risk in counties is compared against the whole country, this change is hard to quantify. Overall many counties saw reduced risk after accounting for the weather.

## Discussion

Emerging diseases have been implicated in millions of honey bee colony losses worldwide^22,23^. Our novel analytical approach clearly demonstrates for the first time that the risk of four of six focal honey diseases is impacted by meteorological conditions, despite being caused by diverse causative agents spanning bacteria, fungi, viruses and mites.

### Meteorological data

We investigated the inclusion of weather data at four temporal scales to consider times that encompassed different aspects of the honey bee life cycle, allowing us to make general observations about colony health. Models containing an average of three months that included the month the apiary was inspected consistently provided the best fit with lower DIC values than other temporal scales (data not shown). This indicates that honey bee colony health is being influenced at a scale that includes the weather at the time of the inspection and the 2 months prior.

### Climatic interactions with honey bee disease

Our chosen meteorological conditions of temperature, rainfall and wind were selected because of their clear influence on honey bee foraging and reproduction. These parameters were found to interact with our focal diseases in different ways. Our data were detrended^24^, and so these relationships are independent of the rise in bee population or in disease prevalence associated with seasonal changes. It is important to note that these harmonic variables are not modelling a process but rather an association with time. For example, the cycle could be linked to the increase in bee populations with season making it easier to detect these diseases. Since the study aimed to model spatio-temporal patterns of risk across counties, any effect that season had on the honey bee population needed to be adjusted. It would be interesting to combine what is learned here about climatic drivers with other known drivers of honey bee disease. For example, a recent European-wide assessment of honey bee health suggested colony mortality and disease presence were reduced by beekeeper education and experience^25^. Future studies could combine beekeeper attributes with meteorological data to assess their relative importance in understanding disease aetiology.

*Varroa* is a considerable problem for beekeepers everywhere due to its commonality and vectoring of other diseases^23^. *Varroa* increased with increasing temperature and reduced with increasing rainfall and wind (Fig. 3). Although *Varroa* reproduction requires active brood rearing, *Varroa* mites move between honey bee colonies by riding on the backs of foraging adult honey bees. Honey bee foraging activity increases as temperatures rise and when rainfall and wind are low^26^. As such, behaviours that lead to the movement of bees between colonies like drifting, where adult bees return to a different colony, and robbing, where foragers steal honey from other weaker colonies, are likely to occur during periods also conducive to foraging. Colony level transmission events, therefore, require fair weather, and most invasions occurred in late summer^27^. Our analysis suggested that the risk of sacbrood virus increased as temperatures rose, which is in line with temporal studies, which suggest sacbrood virus is more likely to occur in warmer months^28^. Chalkbrood is caused by a fungal pathogen, and this brood disease had the opposite relationship with temperature, becoming more likely to occur as temperatures dropped. Although this apparently contradicts laboratory observations that determined the ideal temperature for the growth of the causative agent to be 30 °C^8^, the temperature of the brood nest in a honey bee colony will remain constant, even during cool periods. Fungal pathogens need moisture to replicate and spread, we found no relationship between rainfall and chalkbrood risk, which is surprising.

### Highlighting emerging disease

Our approach allowed us to assess which diseases were increasing in prevalence after accounting for changes due to weather and location. Our analysis highlighted chronic bee paralysis as an emerging disease, which supports previous observations using the same disease dataset^22^. However, our observations indicate that the emergence of chronic bee paralysis is independent of any change in weather patterns highlighting an alternative cause of emergence. Previous studies have indicated an increased risk of chronic bee paralysis associated with certain beekeeping practices such as importation of queens, the scale of beekeeping operation or the addition of pollen traps on colonies^22,29^. Our results indicate a complete understanding of management practices could help understand the recent emergence of chronic bee paralysis, and we can hypothesize that this lack of understanding will be decreased through a governmental interest in CBP.

More worryingly, our analyses indicate that the most damaging honey bee disease globally, varroosis, continued to increase in prevalence during the study period. *Varroa* mites were first recorded in the UK in 1992 and are now endemic within the UK^30^. Our analysis suggests that risk is heightened in the south and west (Fig. 4), which corresponds to the regions where mite resistance to pyrethroid miticides was first reported^30^, and could indicate regional failures in disease control. We do not attempt to investigate interactions between diseases, however, it is interesting to note that sacbrood has a similar pattern of emergence (Fig. 3) and regional risk to varroosis (Fig. 4), despite not being transmitted by *Varroa* mite^31^. *Varroa* infestation is known to reduce the immune competence of honey bees and can help viruses to proliferate in the host^32^. Given the severe direct and indirect impact varroosis can have on honey bee colony survival, it is important we begin to understand the reasons behind the continued emergence.

### Monitoring notifiable diseases

AFB and EFB are the only notifiable honey bee diseases in the UK, and so are the only diseases under a long-running national program of control. AFB was the rarest disease, with only 46 cases being reported in England and Wales in 2016, far lower than other countries (e.g.)^33^. The low number of observations likely contributed to our inability to relate the risk of AFB with any particular driver. AFB is an epidemic disease to England and Wales, with the majority of cases likely being caused by human-assisted movements, such as honey imports, rather than natural spread^34^. AFB is a difficult disease to model as many of the drivers and correlates are hard to identify since AFB epidemics are often opportunistic and random, and cases are often exponentially low. The contribution of anthropomorphic spread have been observed in other regions^11,35^.

EFB was more prevalent than AFB (356 cases in 2016), and our analysis highlighted some interesting features of this endemic disease. EFB risk increased with high levels of rain, weather associated with poor foraging conditions. EFB cases have been linked to colony stress conditions, such as lack of food^4^. Although bees store kilograms of honey during the summer months, they only store a relatively small amount of pollen^36^. Periods of rain or high winds would reduce the opportunity to forage and could add nutritional stress on honey bee colonies, possibly contributing to EFB outbreaks. Interestingly, honey bees resort to brood cannibalism during periods of poor foraging^13^, and this could contribute to a direct increase of within-hive transmission of brood parasites. Certainly, the risk of EFB in the south and west is much reduced once weather variables are accounted for (Fig. 4), suggesting weather is an important factor in the distribution of this disease.

Our analyses provide evidence to suggest that the risk of EFB occurring in England and Wales decreased between 2006 and 2016. A decrease in EFB prevalence was also highlighted by Ref.^34^ when analysing the same dataset between 1994 and 2005 (see Fig. 1 therein). Taken together, these findings suggest that the control program in England and Wales is broadly effective at reducing the total number of EFB cases. However, our data also note some interesting discrepancies in the spatial pattern of risk after accounting for the weather (Fig. 4). Some counties like Lincolnshire, Shropshire, and Herefordshire all appear to have a far higher risk of EFB, suggesting local escape from control measures. Cases of EFB in England and Wales are known to be caused by local clusters of related bacterial ‘sequence types’^37^, which in turn can have different virulence properties^38^. It remains possible that these local escapes in control could be related to differences in the responses of these local bacterial populations to the control methods deployed.

### Regional apiary density

BeeBase is not a register of apiaries but rather a register of apiary inspections, and we found it difficult to estimate apiary density historical data queries. Honey bee colony registration is not mandatory in the UK. Beekeepers can either register directly with BeeBase, NBU inspectors can add beekeepers known to them locally, or sometimes regional beekeeping associations may share membership data. This may lead to regional biases in our apiary density estimates that could explain the large confidence intervals seen in the R INLA fixed effects and the unexpected relationship with the risk of EFB (Fig. 3). Whilst host density is an important factor when considering disease spread, the collection and maintenance of accurate national records is time-consuming and expensive. BeeBase may represent the best proxy for such apiary density data in the UK, but data consistency could be improved using standardised annual data queries to store long-term apiary density data.

### Advantages of INLA

Previous methods used to examine honey bee disease data, such as those used by Ref.^34^, while effective, focus only on the spatio-temporal aspect of honey bees diseases. Their analysis consisted of K-function analysis and kernel density smoothing to determine if disease cases were clustered and spatially distributed. R-INLA has the added advantage of outputting a similar spatio-temporal outlook on honey bee disease in the form of disease risk while also allowing for the drivers of that risk to be considered and outputting the importance of individual drivers. This is not to say that methods such as those employed by Ref.^34^ are ineffective, just that R-INLA now offers a fresh way to deal with complex spatio-temporal disease data.

### Conclusions

Our study provides new insight into the drivers of honey bee disease using modern modelling methods that account for drivers of disease risk and partition the spatio-temporal components of disease transmission. We demonstrate the continued emergence of serious diseases, such as varroosis, even after weather variables have been accounted for. Our data also reveal the general success of long-term statutory control of notifiable diseases, as well as the requirement to further investigate local escapes from control. Climate projections indicate that the UK is likely to see increases in temperature^39^ and rainfall^40^. We highlight how such climatic shifts can contribute to the development of serious diseases such as EFB and varroosis. As such, our work introduces a framework to predict how climate change will impact honey bee diseases, providing a vital step towards the conservation of this important generalist pollinator.

## Methods

### Honey bee health data

Honey bee health inspectors from the National Bee Unit (NBU) of the Animal and Plant Health Agency (APHA) are responsible for conducting approximately 6000 apiary visits in England and Wales per annum. The primary duties of NBU inspectors are to control the statutory notifiable diseases European foulbrood (EFB) and American foulbrood (AFB); to provide husbandry advice to beekeepers; and to control incursions of invasive pests such as the small hive beetle (*Aethina tumida*) and Asian hornet (*Vespa velutina nigrithorax*)^1,41^. In addition, a variety of other attributes are also recorded at each inspection, including the hive type, the presence of non-statutory diseases and colony population characteristics in the form of the number of frames of bees and brood. Apiary inspections mostly occur between April and September to prevent unnecessary stress to the colony. A bee inspector can be called out for various reasons as part of the risk-based inspection program because the beekeeper requested a visit or due to a random apiary inspection^1^.

All inspectors undergo standardised training to undertake honey bee colony health inspections, recognise honey bee diseases, and pass accredited disease diagnosis training before inspecting colonies. However, field symptoms of both foulbrood can vary, and historically every suspected case of AFB and EFB had been confirmed in the laboratory using basic microscopic staining techniques to identify the presence of the causative bacteria in larval/pupal smears^30^. To reduce the time between diagnosis and treatment, commercially available lateral flow immunoassays for detecting AFB and EFB were adopted for the in-field confirmation of foulbrood^42^. From 2006, every suspected case of AFB and EFB was confirmed in the field using a lateral flow immunoassay, and where the lateral flow immunoassay failed to confirm suspect symptoms, a sample was sent to the laboratory for assessment via a larval smear following OIE protocols. In addition, information gathered by the bee inspectors was standardised using a checklist system for ease of entry into the online system.

We built county-level estimates of these measures by collating spatially explicit individual visit data for each year between 2006 and 2016. We assigned disease cases for Varroosis, chalkbrood, sacbrood and CBP using a combination of checkboxes completed during the inspection and by searching the colony notes field for certain keywords related to each disease (Ref.^22^; Table 1). Each putative case was individually checked manually to prevent false positives, so values such as ‘no *Varropsosis* present’ could be excluded. Cases of AFB and EFB were actively recorded, and so data on presence and absence were already available.

**Table 1:** Keywords used to assign diseases cases from honey bee health visit data.

Disease	Key word (disease/disease damage/disease symptoms)
Varroosis	Bad varroa, heavy varroa, severe varroa, dwv, d/w/v, deformed wing, pms, parasitic mite, mite damage, varroa damage, varoasis, varroasis, varroosis, varroa death, died out from varroa, varroa collapse
Chalkbrood	Chalk, chk
Sacbrood	Sac
EFB	EFB is actively looked for during NBU inspections, and therefore data on its presence and absence was already available
AFB	AFB is actively looked for during NBU inspections, and therefore data on its presence and absence was already available
CBP	cpv, cbpv, cbp, bpv, paralysis, shivering, shaking, quivering, trembling, black, shiny, crawling, many dead bees, k-wing

We previously explored possible misdiagnosis of CBP using this method of capturing disease data and clearly showed that the majority of cases identified in the field by NBU bee health inspectors as CBP had high levels of the causative organism chronic bee paralysis virus^22^.

### Apiary density data

BeeBase is a live database that records the colony health at the time of inspections. Registration of honey bee colonies is not compulsory in the UK, and historical data of the number of active apiaries in each county is not routinely recorded. As such, apiary density data over time only exist in the form of historical data queries. We were able to identify two similar data queries that contained information on the number of active apiaries; however, neither query A (2006–2010) nor query B (2009–2016) spanned all years from 2006 to 2016. A Pearsons correlation on the common years indicated a statistic of 0.80, with query B containing ~ 10% more apiaries than query A. Each apiary record was spatially explicit and attributed to county/year. The area of each county was then used to create the apiary density expressed as the apiaries per km^2^ per county per year. To further ensure the data source was not confounding the results, it was included as an independent variable in the analysis.

### Meteorological data

The weather data were obtained from the Centre for Environmental Data Analysis Had-UK dataset, collected by the Met Office. Monthly mean temperatures (°C), rainfall (mm) and average wind speed (m/s) were chosen as meteorological variables because they are highly impactful on honey bee ecology. According to the Had-UK dataset, each data point was paired with weather data from the closest Met Office weather station. The weather variables were then summarised to a monthly county-level scale. As the female honey bee reproductive cycle last approximately 21 days to adulthood and a further 23 days before foraging duties begin, it takes roughly 44 days before an adult bee can begin to collect nectar through foraging^17^. The weather at the time of egg-laying and during larval production, as well as when foraging begins, are likely to have a strong influence on colony health^13,43^. To account for this, meteorological data were investigated at different temporal scales:

1. An average for the month of inspection to account for the weather closest to the timing of the apiary inspection.
2. An average for 1 month before the inspection to account for the development times of any young adult worker bees present at the time of apiary inspection (~ 21 days).
3. An average for 2 months before the inspection to account for any influence weather had on colony condition during the period foragers present at the apiary inspection had been produced, assuming a summer lifespan of 15–38 days^17^.
4. An average of 1–3 above, to account for all the above aspects.

Models were run using each temporal dataset using a progressive modelling strategy (comparing numerous models with only slight variation between them). That allows the models to output which meteorological dataset explains the greatest variation in disease prevalence. Models containing the latter temporal scale (4), effectively an average of three months that included the month the apiary was inspected, consistently provided the best fit with the lowest DIC values (data not shown). As such, this temporal scale was used for all analyses. Finally, the temperature data was passed through a generalised linear model to extract the residuals and remove any effect of season on temperature variation.

### Spatio-temporal analysis

To visualize disease risk over time, the expected number of disease cases within each county each month between 2006 and 2016 was calculated as:

$$E=({D}_{totalcountry}\times {T}_{visitscounty})/{T}_{visitscountry},$$

where ${D}_{totalcountry}$ is the total number of disease cases in the country, ${T}_{visitscounty}$ is the total number of visits in the county and ${T}_{visitscountry}$ is the total number of visits in the country. This calculation accounted for visit bias, whereby an increase in the number of visits may lead to an increase in the number of disease cases recorded^22^.

The inclusion of a seasonal covariate as harmonic transformations of time in the year allows us to factor out the independent contribution of season relative to other covariates. The Conditional Autoregressive (CAR) model is additive insofar as it assesses the independent contribution of each covariate included. By including harmonic variables for seasonality, we effectively assessed the independent contribution of other variables after allowing for the season; this is termed adjustment in medical analyses^24^. All data manipulation was undertaken using the tidyverse package version 1.2.1 in R version 3.6.1^44,45^.

Contagious diseases are dependent on host susceptibility and immune states, but also the proximity of infectious individuals with new potential hosts. Diseases, therefore, inevitably have a spatial component that requires infectious individuals and susceptible hosts to share a location. Using statistical techniques such as logistic regression and generalised linear models is inappropriate as they assume replicates are independent. To analyse patterns of disease effectively, both space and non-independence must be included in the model. Bayesian methods provide a useful initial platform to examine disease data with the strength and robustness residing in the ability to account for uncertainties in the estimates and predictions as well as account for missing data^46,47^. The Besag York Mollie (BYM) model is an autoregressive model that incorporates spatial dependence, meaning cases in one place are assumed to be dependent on other independent risk factors as well as fixed effects associated specifically with the areas in which the response was recorded.

We hypothesised that levels of disease would depend on a series of environmental and demographic drivers predisposed to infection by the different pathogens. We also assumed that since the diseases are contagious to varying extents, proximity to disease would likely lead to local infections. Thus, we anticipated that there would be a temporal and spatial correlation as well as local aggregation of cases of disease. In addition, whilst bee inspectors all received the same initial training to conduct inspections and recognise disease, we appreciated that experience might impact case ascertainment. In order to analyse the epidemiological drivers of disease, we, therefore, needed to address the areas in which bee inspectors operated (typically at a county scale); the demography and environment in the areas where disease was being monitored as well as clustering and spatial and temporal correlation in the incidence of disease^48^. In order to address the problem, we investigated the spatio-temporal patterns of the diseases using area-based ecological regression in the INLA package in R. This is a form of disease mapping that seeks to explain the relative risk of disease in areal units (here counties) in relation to demography, environment spatio-temporal correlation, disease clustering and zero inflation whilst also adjusting for individual bee inspector. The integrated nested laplace approximation (INLA) model is a modern approach to the older and less efficient Markov chain Monte Carlo (MCMC) models. It is a bayesian technique that operates on a class of models known as latent gaussian models^47^. We used the R-INLA package version 21.02.23 set with default priors to analyse the effect of covariates and investigate any spatio-temporal variation. We adopted the R-INLA method because it had the necessary statistical power to analyse spatio-temporal epidemiological data and estimate the effects of dependent variables while still accounting for other spatio-temporal occurrences^49^. The models generate a distribution of estimates for each covariate, and the significance of each can be assessed through the distributions overlap with zero (overlap with zero implying the covariate had no significant effect).

We initially fitted the models with all covariates and then removed those that failed to decrease the Deviance Information Criterion (DIC) by 2, until we found the most parsimonious model explaining the most variation in the spatial–temporal patterns of disease similar to the methods employed by Ref.^49^.

We then completed two sets of risk maps to provide a visual representation of the spatial distribution of the risk of honey bee diseases. Observed and model fitted maps of risk of disease for each disease in each county were produced from the model analyses^46^. The maps were generated using the random marginals from the R-INLA models and are run through the inla.emarginal function to obtain the relative risk for a county compared to the rest of the country. The risk values were rounded to two decimal places to limit the number of values shown. The county boundaries for England and Wales were sourced from the Database of Global Administrative Areas (http://GADM.org).

## Supplementary Information

Supplementary Table S1.

## Data Availability

Visit data were obtained under a data confidentiality agreement from the Animal and Plant Health Agency (contact enquiries@apha.gsi.gov.uk). The weather data is open source from the CEDA visit https://www.ceda.ac.uk/services/ceda-archive/ for more details.
