# Supplementary material for: Identifying the climatic drivers of honey bee disease in England and Wales
Source: Sci Rep. 2021 Nov 9;11:21953. doi: 10.1038/s41598-021-01495-w (PMC8578631; doi:10.1038/s41598-021-01495-w)
Supplement: Supplementary file 1 — Supplementary Table S1. [file 41598_2021_1495_MOESM1_ESM.pdf]

Supplementary Information

Identifying the climatic drivers of honey bee disease in England and Wales

Ben W. Rowland[1]*, Steven P. Rushton[1], Mark D.F. Shirley[1], Mike A. Brown[2] and Giles E. Budge[1]

[1]School of Natural and Environmental Sciences, Newcastle University, Newcastle upon Tyne, Tyne and Wear NE1 7RU, UK.

[2]National Bee Unit, Animal and Plant Health Agency, Sand Hutton, York YO41 1LZ, UK.

Supplementary Table S1 *Results of the R-INLA spatio-temporal models for EFB, AFB, CBP, varroosis, chalkbrood and sacbrood containing all significant covariates after the models were improved using the DIC values. Significant values are determined if both the 2.5% and 97.5% confidence intervals are positive or negative (ie does not include 0). These are represented in bold here.*

Disease	Covariate	Mean	SD	2.5%	97.5%
EFB	Intercept	0.520	0.447	-0.358	1.399
EFB	Mean Temp	-0.015	0.064	-0.141	0.111
**EFB**	**Rain**	**0.011**	**0.002**	**0.006**	**0.015**
**EFB**	**Cosine**	**-1.999**	**0.122**	**-2.238**	**-1.760**
**EFB**	**Sine**	**-0.304**	**0.082**	**-0.465**	**-0.144**
**EFB**	**Density**	**-2.702**	**1.236**	**-5.132**	**-0.279**
**EFB**	**Data Source**	**0.860**	**0.224**	**0.420**	**1.299**
**EFB**	**Time**	**-0.008**	**0.003**	**-0.015**	**-0.002**
**AFB**	**Intercept**	**0.111**	**0.033**	**0.046**	**0.177**
**AFB**	**Cosine**	**-0.082**	**0.031**	**-0.143**	**-0.020**
AFB	Sine	-0.010	0.020	-0.050	0.030
**CBP**	**Intercept**	**-0.196**	**0.060**	**-0.314**	**-0.078**
**CBP**	**Time**	**0.007**	**0.001**	**0.005**	**0.008**
**varroosis**	**Intercept**	**5.640**	**1.599**	**2.502**	**8.779**
**varroosis**	**Mean Temp**	**0.388**	**0.180**	**0.035**	**0.741**
**varroosis**	**Rain**	**-0.014**	**0.007**	**-0.028**	**-0.001**
**varroosis**	**Wind**	**-0.788**	**0.389**	**-1.551**	**-0.025**

**varroosis**	**Cosine**	**-1.085**	**0.362**	**-1.795**	**-0.376**
**varroosis**	**Sine**	**-1.554**	**0.286**	**-2.116**	**-0.993**
**varroosis**	**Time**	**0.060**	**0.004**	**0.051**	**0.069**
chalkbrood	Intercept	0.053	0.411	-0.753	0.858
**chalkbrood**	**Mean Temp**	**-0.616**	**0.200**	**-1.008**	**-0.224**
**chalkbrood**	**Cosine**	**-7.474**	**0.403**	**-8.265**	**-6.684**
**chalkbrood**	**Sine**	**-1.517**	**0.260**	**-2.028**	**-1.006**
**chalkbrood**	**Time**	**0.058**	**0.005**	**0.049**	**0.068**
sacbrood	Intercept	-1.363	1.010	-3.346	0.618
sacbrood	Mean Temp	0.198	0.111	-0.020	0.416
sacbrood	Rain	0.008	0.004	0.000	0.016
**sacbrood**	**Wind**	**-0.495**	**0.236**	**-0.959**	**-0.032**
**sacbrood**	**Cosine**	**-2.977**	**0.219**	**-3.406**	**-2.548**
**sacbrood**	**Sine**	**-0.877**	**0.175**	**-1.221**	**-0.535**
**sacbrood**	**Density**	**4.235**	**1.691**	**0.916**	**7.556**
**sacbrood**	**Time**	**0.043**	**0.004**	**0.034**	**0.052**
